# The Interplay between Liver and Adipose Tissue in the Onset of Liver Diseases: Exploring the Role of Vitamin Deficiency

**DOI:** 10.3390/cells13191631

**Published:** 2024-09-30

**Authors:** Ivan Tattoli, Aimee Rachel Mathew, Antonella Verrienti, Lucia Pallotta, Carola Severi, Fausto Andreola, Virve Cavallucci, Mauro Giorgi, Mara Massimi, Lapo Bencini, Marco Fidaleo

**Affiliations:** 1 Oncology General Surgery, Azienda Ospedaliero Universitaria Careggi, 50139 Florence, Italy; ivan.tattoli70@gmail.com (I.T.); bencinil@aou-careggi.toscana.it (L.B.); 2 Department of Biology and Biotechnologies “Charles Darwin”, Sapienza University of Rome, 00185 Rome, Italy; aimeerachel.mathew@uniroma1.it (A.R.M.); mauro.giorgi@uniroma1.it (M.G.); 3 Department of Translational and Precision Medicine, Sapienza University of Rome, 00161 Rome, Italy; antonella.verrienti@uniroma1.it (A.V.); lucia.pallotta@uniroma1.it (L.P.); carola.severi@uniroma1.it (C.S.); 4 Liver Failure Group, Institute for Liver and Digestive Health, Royal Free Hospital, University College London, London NW3 2PF, UK; f.andreola@ucl.ac.uk; 5 Department of Translational Medicine and Surgery, Università Cattolica del Sacro Cuore, Fondazione Policlinico Universitario “A. Gemelli” IRCCS, 00168 Rome, Italy; virve.cavallucci@unicatt.it; 6 Department of Life, Health and Environmental Sciences, University of L’Aquila, 67100 L’Aquila, Italy; mara.massimi@univaq.it; 7 Research Center for Nanotechnology for Engineering of Sapienza (CNIS), Sapienza University of Rome, 00185 Rome, Italy

**Keywords:** liver, adipose tissue, vitamin deficiency, liver-adipose tissue axis, MASLD, NAFLD

## Abstract

The deficiency of vitamins, a condition known as “hidden hunger”, causes comprehensive pathological states. Research over the years has identified a relationship between liver diseases and hypovitaminosis or defects in vitamin metabolism. The exact mechanisms remain elusive; however, the crucial involvement of specific vitamins in metabolic functions, alongside the reclassification of liver disease as metabolic dysfunction-associated steatotic liver disease (MASLD), has prompted researchers to investigate the potential cause-effect dynamics between vitamin deficiency and liver disease. Moreover, scientists are increasingly investigating how the deficiency of vitamins might disrupt specific organ crosstalk, potentially contributing to liver disease. Although the concept of a dysmetabolic circuit linking adipose tissue and the liver, leading to liver disease, has been discussed, the possible involvement of vitamin deficiency in this axis is a relatively recent area of study, with numerous critical aspects yet to be fully understood. In this review, we examine research from 2019 to July 2024 focusing on the possible link between liver-adipose tissue crosstalk and vitamin deficiency involved in the onset and progression of non-alcoholic fatty liver disease (NAFLD). Studies report that vitamin deficiency can affect the liver-adipose tissue axis, mainly affecting the regulation of systemic energy balance and inflammation.

## 1. Introduction

Vitamins are essential micronutrients for optimal growth and development, required in smaller quantities than macronutrients. While the immediate impacts of undernourishment related to macronutrients, i.e., carbohydrates, proteins, and lipids, are visible, vitamin deficiencies may not be immediately apparent, leading to ‘hidden hunger’. This issue affects over two billion people worldwide, with a higher prevalence in low- and middle-income countries where affordable food staples and dietary diversity are limited [1]. Insufficient nutritional intake is just one aspect of the broader picture of vitamin deficiency. Numerous health conditions can compound this issue, including, but not limited to, a heightened incidence of gastrointestinal infections, intestinal malabsorption (encompassing inflammatory diseases like celiac disease and gastric bypass surgery), intestinal microbiota alterations, known as dysbiosis (resulting from several factors, including antibiotic therapy), inadequate exposure to sunlight, and organ failure (kidney and liver, pancreatic disease) [2,3,4,5]. Additionally, specific autoimmune conditions targeting the pathways responsible for micronutrient absorption can contribute to their deficiency. For instance, in the autoimmune disease pernicious anemia, anti-intrinsic factor antibodies inhibit the binding of the intrinsic factor for vitamin B12, preventing the absorption of the vitamin at the terminal ileum [6]. Finally, stress-related behaviors, including smoking, alcohol, and coffee consumption, contribute significantly to the complexities of vitamin deficiency onset [7,8,9,10,11,12]. Over the years, several studies have found evidence of a connection between liver diseases and hypovitaminosis. However, there are still gaps in understanding the underlying basis of this correlation [13]. There is a mutual cause-effect link between vitamin deficiencies and hepatic diseases. On the one hand, vitamin deficiency associated with liver fibrosis and steatosis could be an etiological factor at the outset of the disease. This hypothesis is supported by the observation that an inadequate dietary intake of vitamins E, D, and A is related to a higher prevalence of non-alcoholic fatty liver disease (NAFLD) and non-alcoholic steatohepatitis (NASH) [14]. On the other hand, the outcome of the progressive deterioration of liver function causes a failure of proper absorption and storage and a disturbance of metabolic intermediaries of vitamins, thus contributing to deficiencies, including vitamins A and K. For instance, altered biliary secretion in the intestinal tract compromises the absorption of vitamins A, D, E, K, C, and group B [15]. While the link between hypovitaminosis and liver disease has emerged in recent years and mechanisms are being elucidated, there remains a lack of specific guidelines and reference values for patients’ blood levels. Consequently, discussions about hypovitaminosis related to liver disease are often reported in more general terms as a “deficiency” when compared to healthy individuals [13,16,17].

NAFLD is the most common chronic liver disease worldwide, affecting 20–30% of the general population [13]. It presents a wide range of liver damage, spanning from mild steatosis to severe NASH, with or without accompanying fibrosis, cirrhosis, and hepatocellular carcinoma (HCC). In numerous countries, it ranks as the primary cause of HCC, frequently diagnosed in advanced stages with an unfavorable prognosis [18] with a heavy clinical, social, and economic worldwide burden [19,20]. There are no approved drugs for NAFLD treatment. Currently, lifestyle modifications, particularly dietary changes, represent the primary approach to preventing the progression of NAFLD and its potential complications. However, in some cases, this approach may prove ineffective in halting the progression of pathogenesis [21,22]. Recently, there has been a need to rename liver diseases that lead to steatosis (now collectively referred to as steatotic liver disease, SLD) to provide a more accurate description [23]. These new labels, metabolic dysfunction-associated steatotic liver disease (MASLD) and metabolic dysfunction-associated steatohepatitis (MASH), further emphasize the primarily metabolic nature and highlight the complex interplay between metabolic abnormalities and liver pathology [24,25]. Some other authors also use the terminology metabolic-associated fatty liver disease (MAFLD) [26]. Although the new terminologies MASLD and MASH may almost overlap with the previous definitions of NAFLD and NASH, respectively, it should be noted that the MASLD definition includes SLD cases with specific cardiovascular risks, while cases without these risks are referred to as cryptogenic SLD. Thus, a direct conversion between the old and new nomenclature is not possible. For instance, in a large population-based study (17,595 subjects) conducted in 2024, the authors reanalyzed NAFLD cases according to the new nomenclature. It emerged that the vast majority of these NAFLD patients met the criteria for MASLD, but a small percentage (3.5%) fell into the cryptogenic SLD group [27]. Taking this into account, although the new terminology is more accurate compared to the previous one, we believe that altering the nomenclature used by the researchers we have cited could cause confusion; therefore, we have retained the original nomenclature in this review.

The metabolic aspect of SLD is further underscored by the “theory of multiple parallel hits,” which suggests that various factors act synergistically and at different levels in a parallel and non-sequential manner to generate NAFLD. These factors include: (i) adipokines, such as leptin, adiponectin, ghrelin, resistin, retinol-binding protein (RBP) 4, visfatin, chemerin, and adipocyte fatty acid-binding protein (AFABP) [28]; (ii) gut dysbiosis; (iii) increased gut permeability (leaky gut) [29,30]; and (iv) exposure to environmental agents (food, air, soil, and water pollution) [31]. They interact with each other in genetically predisposed individuals, in addition to well-known factors like insulin resistance and oxidative stress, and intricately influence metabolic processes [32,33]. These observations highlight the complexity of NAFLD while also providing evidence of its multi-organ nature. This aspect has been well emphasized in the new SLD terminology, where, for example, obesity is one of the inclusion criteria for MASLD, distinguishing it from a similar condition, i.e., cryptogenic SLD [27]. Moreover, it is important to emphasize that metabolic dysfunctions like obesity are not merely associative phenomena; clinical data have also underscored the fundamental role of dysregulation in the liver-adipose tissue axis in SLD (see Table 1 for references).

The well-known role of vitamins in the metabolism, alongside the recent concept of MASLD, definitively underscores their significance in the illness. Moreover, because vitamin deficiency affects not only the liver but also the entire body, scientists are increasingly exploring how such deficiencies may disrupt specific organ axes, potentially contributing to liver disease. The relationship between liver diseases, adipose tissue, and vitamin deficiency is a relatively recent area of focus, with many crucial points still to be uncovered. In this review, we analyze research from the past five years (2019–2024) and report potential molecular and cellular mechanisms involved in the initiation and progression of NAFLD, focusing on the axis of liver-adipose tissue interaction and vitamin deficiency.

## 2. Liver Disease: The Link between Adipose Tissue and the Liver

The liver presents a well-organized cellular architecture, which mirrors its complex and wide variety of functions. The liver is typified by the parenchymal cells (hepatocytes) and non-parenchymal cells. The latter include resident macrophages (Kupffer cells, KCs), hepatic stellate cells (HSC), lipocytes cells, and the sinusoidal intrahepatic lymphocytes (IHL). A signaling network connects parenchymal and non-parenchymal cells, maintaining hepatic functions under homeostatic conditions [34,35]. Alterations in hepatic and organ-related metabolic processes and pathways lead to changes in the hepatic histological spectrum, progressing toward chronic disease, which is accompanied by metabolically altered hepatocytes, inflammation, and fibrosis [36]. For instance, the progression of liver disease is associated with the capillarization of sinusoids, which affects their permeability. This impairs the passage of molecules such as nutrients, lipoproteins, and toxins, leading to decreased efficiency in the exchange of lipids and metabolic substances between the blood and hepatocytes. Consequently, this exacerbates liver metabolic dysfunction (e.g., lipid metabolism) and hinders the passage of drugs, complicating liver targeting [37,38].

Adipose tissue is composed of adipocytes and stromal vascular cells. Adipocytes can be distinguished into white and brown, which form white adipose tissue (WAT) and brown adipose tissue (BAT), respectively. The WAT stores energy through triacylglycerols (TAGs), which are hydrolyzed into glycerol and free fatty acids (FFAs). The glycerol undergoes gluconeogenesis in the liver, while FFAs are released into the blood and are either oxidized by β-oxidation in various tissues or converted to ketone bodies in the liver. BAT is conferred with a thermogenic capacity called adaptive or non-shivering thermogenesis (NST), which refers to a mechanism by which the body generates heat without shivering [39].

**Table 1:** Summary of Key Findings from Relevant Articles Regarding the Involvement of Vitamins in Impairing the Crosstalk Between Adipose Tissue and the Liver in Non-Alcoholic Fatty Liver Disease (NAFLD) Pathogenesis (2019–2024). Papers were identified as eligible from the PubMed database search using the keywords “liver”, “adipose”, and “vitamin” in abstracts from 2019 to 2024, and subsequently selected through manual review. Studies involving multivitamin supplements or treatments combined with vitamins were excluded from this table.

Vitamin	Ref.	Year	Highlights
A	[40]	2022	β-carotene 15,15′-monooxygenase 1 (BCMO1) is a crucial enzyme that converts β-carotene into vitamin A. It is considered an important regulator of lipid metabolism in adipocytes and has the effect of preserving liver functions.
	[41]	2022	Retinoic acid (RA) reveals a therapeutic effect on NAFLD by increasing fatty-acid (FA) oxidation in the liver and thermogenesis and white adipose tissue (WAT) browning in adipose tissue.
	[42]	2023	Adipose-derived mesenchymal stem cells (ADMSCs) have shown significant therapeutic potential in treating liver fibrosis by upregulating the expression of various genes to promote retinol metabolism.
	[43]	2024	Retinol-binding protein 4 (RBP4) produced by visceral adipocytes contributes to the transport and mobilization of hepatic retinol storage. In a high-fat diet (HFD), increased plasma retinol levels are correlated with high serum RBP4 levels, which could be associated with an increased risk of NAFLD since RBP4 contributes to free fatty acid (FFA) mobilization from adipose tissue to the liver.
B3	[44]	2019	High niacin supplementation plays a pivotal role in the human NAFLD pathogenesis by inhibiting lipolysis in the adipose tissue of humans, which thereby decreases the amount of free-flowing FAs into the liver.
	[45]	2021	Nicotinamide (NAM) administration increases the mitochondrial β-oxidation of FAs in brown adipose tissue (BAT), and triggers a browning process in WAT, which enhances its energy expenditure. NAM also keeps a check on hepatic steatosis.
	[46]	2022	N1-Methylnicotinamide (mNAM) induces lipolysis in adipose tissue and gluconeogenesis in hepatocytes under physiological conditions, and releases ketone bodies and glucose as metabolic substrates in skeletal muscle.
	[47]	2024	mNAM decreases hepatic lipid accumulation and reduces inflammation in WAT.
	[48]	2022	Supplementation with nicotinamide riboside (NR) exerted an anti-obesity effect and prevented the development of inflammation and fibrosis in the WAT of old, but not young female mice with diet-induced obesity, protecting the liver from obesity effects.
	[49]	2022	NR reduces lipogenesis in the liver and increases lipolysis in WAT, in high-fructose and high-fat induced models, suggesting a possible therapeutic application in lipid metabolic disorders.
B5	[50]	2022	Pantothenate enhances BAT energy expenditure in an uncoupling protein 1 (UCP1)-dependent manner and reduces adiposity and, thereby indirectly, hepatic steatosis.
B7	[51]	2022	In rats, high doses of dietary biotin intake can activate FA oxidation due to the increased hepatic β-oxidation, which, in turn, may contribute to the reduction in the hepatic triglyceride (TG) concentration and WAT weight.
B9	[52]	2023	There is a relationship between folic acid, gut microbiota, liver and adipose tissue.
C	[53]	2020	Vitamin C administration at a medium dose is beneficial for prophylaxis and therapy of HFD-induced NAFLD, while at a low dose it prevents the development of HFD-induced NAFLD and aids in its management. Moreover, it could prevent HFD-fed mice from weight and visceral fat gain. Conversely, a high dose may be risky.
	[54]	2022	Vitamin C modulates hepatic expression and secretion of growth factor 21 (Fgf21) which, in turn, enhances BAT thermogenesis and regulates lipid metabolism.
	[55]	2021	Supplementation of vitamin C can dysregulate WAT hyperplasia and hepatic steatosis by reversing the hypermethylation due to Tet1 haploinsufficiency, resulting in FA oxidation lipolysis and thermogenic upregulation.
D	[56]	2020	In humans and mice, obesity suppresses the vitamin D 25-Hydroxylase (Cytochrome P450 2R1, CYP2R1) in mouse liver and BAT and in human subcutaneous WAT (sWAT), leading to vitamin D deficiency.
	[57]	2022	Vitamin D deficiency promotes NAFLD due to adipose tissue metabolism dysfunction, thereby altering the crosstalk between the liver and adipose tissue.
	[58]	2022	Vitamin D deficiency promotes NAFLD by trigging WAT-associated macrophage infiltration and secretion of bioactive inflammatory adipokines and resulting in extracellular matrix (ECM) remodeling, which ultimately causes fibrosis.
	[59]	2020	Supplementation of vitamin D reduces WAT inflammation by downregulating the related markers (such as *Mcp1* and *Ccl5*) and reduces de novo lipogenesis by downregulating the FA synthase (*Fasn*) and acetyl-CoA carboxylase 1 (*Acaca*), which, in turn, decreases the hepatic lipid droplets (LDs) in the liver.
	[60]	2021	The high-fat/sucrose-induced inflammation in inguinal adipose tissue and hepatic steatosis was reduced by the synergetic effect due to the combination of physical exercise and vitamin D supplementation, leading to a reduction of inflammation in WAT and in the liver.
	[61]	2022	Supplementation of vitamin D improved the HFD-induced weight gain, hepatic steatosis, serum lipid profile, degree of inflammation, and serum adipokine levels.
	[62]	2023	Calcifediol is considered suitable for all patients with vitamin D deficiency since it is better absorbed, has higher biological activity, and is less prone to sequestration in adipose tissue and may be preferred over vitamin D3 for patients with obesity, liver disease, and malabsorption and those who require a rapid increase in 25-hydroxyvitamin D3 concentrations.
E	[63]	2021	Supplementation of α- and γ-tocopherol in the ratio 1:5 reduces and attenuates adipocyte enlargement, hepatic steatosis, and metabolic inflammation (induced by HFD).
	[64]	2022	Prolonged vitamin E supplementation can dysregulate interrelated miRNA profiles in the liver and WAT through negative feedback regulation, negatively impacting lipid metabolism in both the liver and WAT.
K	[65]	2024	In an HFD-induced NAFLD mouse model, vitamin K2 reduced the visceral fat burden without reducing the lean mass and free body fluid, and prevented hepatic steatosis, inflammation, and fibrosis.

WAT can be distinguished into: visceral WAT (vWAT), deposited in gluteal, abdominal, and femoral regions; and subcutaneous WAT (sWAT), present in gonadal (including epididymal fat, eWAT, located around the epididymis in male animals), mesenteric, epicardial, omental, and retroperitoneal regions with specific functions [66,67,68,69,70,71,72,73,74]. BAT depots are concentrated in the supraclavicular, cervical, paravertebral, perirenal, and mediastinal regions with specific functions [66,75,76,77,78,79,80,81,82,83]. Table 2 reports the location and function of WATs and BATs. Since genetic background, epigenetic differences, ethnicity, aging hormonal alteration, and medications have a distinct influence on body region distribution; each fat depot contributes differently as a metabolic and endocrine organ, leading to different levels of metabolic disorder or therapeutic responses [67].

**Table 2:** White adipose tissue (WAT) and brown adipose tissue (BAT) location and functions.

Adipose Tissue		Functions	References
White Adipose Tissue (WAT)	Femoral-gluteal WAT	Protects against insulin resistance and cardiovascular diseases (CVDs)	[67]
	Subcutaneous and abdominal WAT (sWAT)	Associated with insulin resistance, metabolic syndrome, type 2 diabetes mellitus (T2DM), and CVDs	[68]
	Gonadal (gWAT) (epididymal fat in males, eWAT, and periovarian fat in females)	Regulates gametogenesis via modulation of neuroendocrine signaling and lipid deposition and supports lipid metabolism	[69]
	Mesenteric Adipose Tissue (MAT)	Lipid storage, upholding the intestinal barrier, regulation of immune function, and intestinal flora intestinal permeability	[70,71]
	Epicardial Adipose Tissue (EpAT)	EpAT has physiological and pathological properties that vary depending on its location. It can be highly protective for the adjacent myocardium through dynamic brown fat-like thermogenic function and harmful via paracrine or endocrine secretion of pro-inflammatory and profibrotic cytokines	[72]
	Omental WAT (oWAT)	It presents immunomodulatory functions	[73]
	Retroperitoneal WAT (rWAT)	It has positive effects on cardiovascular, metabolic, inflammatory, and hormonal changes induced in high-fat conditions	[74]
Brown Adipose Tissue (BAT)	Paravertebral and supraclavicular BAT (scBAT)	Protect against hypothermia to maintain optimal function and nerve conduction in the central and autonomic nervous system and immune functions. Thermogenic and cardiometabolic function	[75,76,77,78]
	Cervical BAT	Associated with cardiometabolic homeostasis depending on gender and metabolic status	[79]
	Perirenal BAT (PRAT)	It influences metabolic, renal, and cardiovascular homeostasis, and controls the plasticity of brown/white adipose phenotypes	[80,81]
	Mediastinal BAT (MAT)	Prognostic biomarker of cardiovascular diseases	[82,83]

**Figure 1:**
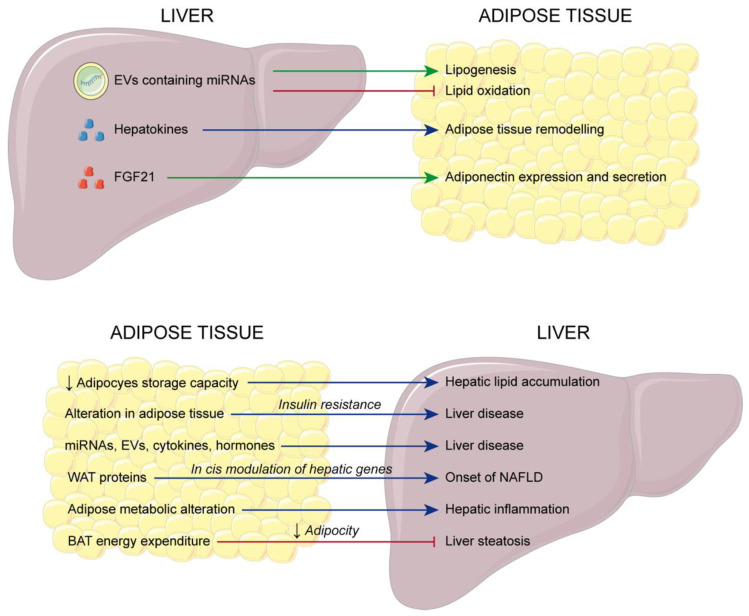
A Proposed Dysmetabolic Circuit Between Adipose Tissue and Liver Leading to Liver Disease. This figure illustrates a potential cause-effect dysmetabolic circuit between adipose tissue and the liver, contributing to the development of steatotic liver disease (SLD). See the text for further details. This image was created using Inkscape. → Activation/Induction/Stimulation (Blue arrows are used when multiple mechanisms are involved; specific mechanisms are noted on the arrow itself). −| Inactivation/Inhibition/Blockage.

A potential cause-effect dysmetabolic circuit between adipose tissue and the liver that results in liver disease has long been discussed (Figure 1). Specifically, lipotoxicity of adipose tissue, together with alterations in gut microbial functions, contribute to the evolution of inflammation and fibrosis in NAFLD [84]. Insulin resistance, resulting from increased secretion of pro-inflammatory cytokines, adipokines, and lipids from visceral adipose tissue, heightens the mobilization of FA, leading to hepatic fat accumulation. Consequently, hepatic steatosis causes endoplasmic reticulum stress, mitochondrial dysfunction, and oxidative stress, ultimately resulting in increased cytokine production and subsequent inflammation [85]. Details of molecular mechanisms have been built up more recently. Interestingly, BAT and WAT have different effects on the liver, preventing and contributing to the onset and progression of NAFLD, respectively [86]. It has been demonstrated that adipose tissue controls hepatic metabolism by the secretion of miRNAs, extracellular vesicles, cytokines, and hormones, whose dysfunction triggers energy- and inflammatory-dependent signaling pathways that are responsible for NAFLD onset and further transition from NAFLD to NASH [87,88,89]. Moreover, adipose-liver crosstalk is supported by a gene co-expression network mediated by adipose WAT genes (*COL6A2*, *CCDC80*, *SOD3*), whose proteins secreted into the serum can modulate in cis hepatic genes responsible for the onset of NAFLD. These proteins can be identified as serum biomarkers [90,91]. In addition, impaired upregulation of genes involved in adipogenesis in WAT, i.e., *SOCS3*, *DUSP1*, *SIK1*, and *GADD45B*, determines a lower storage capacity in adipocytes of lipids and can cause hepatic lipid accumulation, contributing to NAFLD development and progression [92]. Furthermore, since metabolic alterations found in adipose tissue hypertrophy exacerbate hepatic inflammation and dysfunctional metabolism, adipose-related metabolites could play a pivotal role in the crosstalk between adipose tissue and the liver in health and disease conditions [93]. On the other hand, the liver modulates adipocyte metabolism under lipid overload by making extracellular vesicles (EV) containing miRNAs (e.g., let-7e-5p and miR-210-3p) that upregulate lipogenesis and inhibit lipid oxidation, leading to a healthier condition [94]. Moreover, hepatokines, secreted by the liver, have several effects on adipose tissue remodeling and the development of obesity [95]. The liver can also regulate glucose and lipid homeostasis by inducing an increase in the expression and secretion of adiponectin from adipose tissue [96].

**Figure 2:**
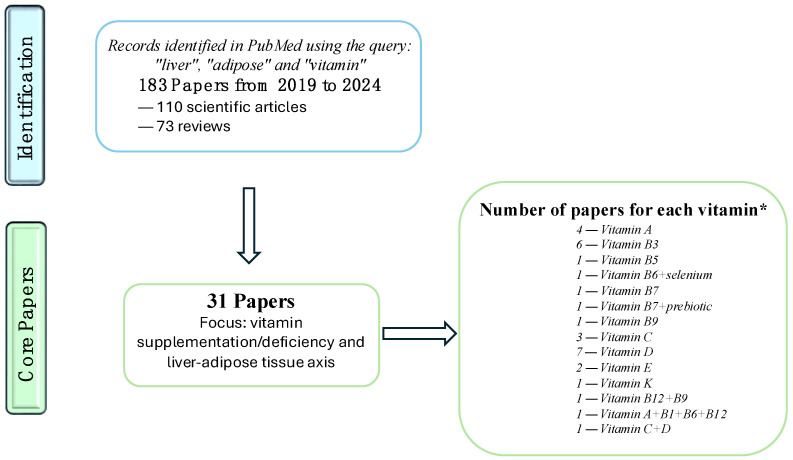
Flowchart of Systematic review. * Studies involving multivitamin supplements or treatments combined with vitamins were excluded from Table 1.

The physiological role of vitamins has been well characterized at the hepatic level, while less information is available regarding their role in adipose tissue. Similarly, vitamin deficiency and its effects have been well characterized in the liver (including in relation to the onset of liver diseases) and less so in adipose tissue. Although the cause-effect dysmetabolic circuit between adipose tissue and the liver is quite well known, as mentioned earlier, the effect of vitamin deficiency/supplementation on this circuit is truly limited. Here, we conducted a scoping review to investigate the interplay between the liver, adipose tissue, and vitamins by querying the PubMed database using the keywords “liver”, “adipose”, and “vitamin” in abstracts from 2019 to 2024. Initially, our search yielded 183 articles. However, after screening the abstracts for their relevance to the specific relationship between the liver, adipose tissue, and vitamins, only 31 articles were considered pertinent. These articles were further analyzed to extract key information, including the reference, publication year, type of vitamin investigated, and the main conclusions drawn. To avoid a misunderstanding of the possible effects in the case of multivitamin treatment or treatments combined with vitamins, we did not consider the articles studying more than one vitamin or combined treatment [97,98]. We also included reviews that report data from original articles published before the considered time window, as their inclusion could allow us to indirectly incorporate the most relevant information obtained prior to 2019. The findings are summarized in Figure 2 and Table 3, Table 4, Table 5, Table 6, Table 7 and Table 8. Vitamin D emerges as the most frequently studied, with 10 articles dedicated to its role in this context. Based on the results of the literature analysis, we proceeded to delve into the topic by examining the role of each vitamin.

## 3. Vitamin A

The human body relies on dietary intake for vitamin A since it cannot synthesize it internally. Animal-based foods provide preformed vitamin A, found in the form of retinyl esters and retinol, which is essential for vision, skin maintenance, and human development [99]. Conversely, fruits and vegetables offer pro-vitamin A carotenoids, such as α-carotene, β-carotene, or β-cryptoxanthin. Upon absorption by enterocytes, these pro-vitamin A carotenoids convert into retinol and retinyl esters [100]. Retinol and retinol derived from preformed vitamin A undergo esterification into retinyl esters, which are then packaged into chylomicrons (CMs) for secretion into the lymphatic system [101]. Additionally, retinol can undergo conversion into various derivatives known as retinoids, each possessing distinct biological activities. Among these, retinaldehyde (Ral) and retinoic acid (RA) are the primary biologically active molecules, regulating numerous cell-specific physiological processes in peripheral tissues. Ral and RA exert their biological effects by binding to specific nuclear receptors known as retinoic acid receptors (RARs) and retinoid X receptors (RXRs). These receptors are members of the steroid hormone receptor superfamily and function as ligand-activated transcription factors [102,103].

The liver plays a central role in the postprandial storage and metabolism of vitamin A (retinol). Indeed, 70% of chylomicron retinoids and carotenoids are cleared from the circulation by the liver, and just 25–30% from extrahepatic tissues. In hepatocytes, retinyl esters are hydrolyzed in retinol that can be secreted or transferred into HSC, where more than 95% are primarily esterified in palmitate esters, contained in lipid droplets (LDs) as energy reservoirs [104]. The mobilization of retinol to target tissues necessitates the hydrolyzation of stored retinyl esters in HSCs into retinol. This retinol is then transferred into hepatocytes, where it binds to RBP4, which functions as a specific carrier for retinol in the circulation [105], where it is released into the bloodstream in a complex with transthyretin (TTR) [106]. It is essential to note that RBP4 is not solely secreted by the liver; adipose tissue also contributes to its secretion, indicating its role as an important player in retinol storage and mobilization [107].

In adipose tissue, retinol bound to RBP4, and retinyl ester and β-carotene present in CMs are internalized [106]. In adipocytes, vitamin A plays a crucial role as a regulator of their differentiation and thermogenic programming. Specifically, RA serves to prevent metabolic dysfunction through genetic DNA methylation, contributing differentially to adipose tissue homeostasis. It prevents de novo adipogenesis, thereby impeding lipid accumulation in both WAT and BAT [33], ultimately mitigating metabolic dysfunction.

Evidence for the involvement of vitamin A in the regulation of the crosstalk between adipose tissue and liver is provided by the murine β-carotene 15,15′-monooxygenase 1 (*Bcmo1*) knockout model. Bcmo1 is a crucial enzyme responsible for converting β-carotene into vitamin A (Table 3). Differentially expressed gene (DEG) analysis between wild-type females and *Bcmo1* knockout females, both under a β-carotene diet, reveals that Bcmo1 plays a vital role in maintaining vitamin A and lipid metabolism in adipocytes through pathways involving peroxisome proliferator-activated receptor α (Pparα), ATP-citrate lyase (Acly), and fatty acid-binding protein 5 (Fabp5). These pathways represent a central hub that gives rise to a complex signaling network, regulating adipocyte lipid metabolism to preserve liver function in a healthy state and prevent steatosis-induced liver failure [40]. Furthermore, β-carotene, both dependently and independently from its active forms (retinal and RA), exhibits thermogenic activity inducing uncoupling protein 1 (UCP1)-dependent or independent NST, probably acting on both brown and white adipocytes [108,109,110], contributing more efficiently to lipolysis and thereby suggesting a positive impact on liver disease. This occurs through the activation of the β3-adrenergic receptor (β3-AR) and cyclic AMP (cAMP) for either UCP1-dependent or independent NST, as well as the α1-adrenergic receptor (α1-AR) pathway for UCP1-independent thermogenesis [111], contributing more efficiently to lipolysis and thermogenesis, thereby suggesting a positive impact on liver disease. Moreover, in high-fat diet (HFD) mice, RA demonstrates a therapeutic impact on NAFLD by boosting the transcription of fatty acid oxidation genes (*Cpt1B*, *Acox1*, *Pgc1*) and thermogenesis-related genes (*Ucp1* and *Pparγ*). Surprisingly, this effect is achieved by activating RARs, primarily in WAT rather than the liver [41] (Table 3). The potential beneficial role of adipose tissue as a trigger for liver disease improvement is further supported by studies demonstrating that transplantation of adipose-derived mesenchymal stem cells (ADMSCs) can regulate the expression of genes associated with bile acid homeostasis and vitamin A metabolism. This modulation leads to the mitigation of NAFLD, underscoring the therapeutic promise of ADMSCs [42]. Moreover, in HFD mice RA demonstrates a therapeutic impact on NAFLD, primarily through the activation of thermogenesis and induction of WAT browning rather than its effect on the liver. Indeed, RA increases the transcription of fatty acid oxidation and thermogenesis-related genes activating RARs, primarily in WAT [41]. Interestingly, it has been noted that the distribution of adipose tissue, particularly increased visceral adiposity, correlates with serum vitamin A depletion in women, even when meeting recommended dietary intake levels. However, the precise mechanisms underlying this association remain partially understood [112].

Although low levels of vitamin A are associated with the onset of metabolic liver diseases, it has been observed that chronic elevation of vitamin A, primarily due to increased levels of the vitamin A carrier RBP4, is also associated with the progression and severity of NAFLD [43]. At the hepatic level, an excess of RBP4 impairs mitochondrial lipid oxidation [113] and may promote the generation and accumulation of reactive oxygen species (ROS) via the reduced-form nicotinamide adenine dinucleotide phosphate (NADPH) oxidase 2 (NOX2) in KCs, promoting their M1-like polarization, and thus inflammation [114] (Table 3). Moreover, high levels of RBP4 promote adipose inflammation, leading to hepatic steatosis and insulin resistance, thus highlighting a possible relationship between vitamin A and the adipose-liver axis at an early stage of liver disease [115,116].

**Table 3:** Key Mechanisms of Vitamin A’s Role in the Liver, Adipose Tissue, and the Liver-Adipose Tissue Axis. Gene and protein acronyms are reported at the end of the manuscript.

Compound	Effects on Liver	Effects on Adipose Tissue	Mechanisms	Models	Treatment	Reference
Retinoic acid (RA)	Reduction in fat deposition, and hepatic triglyceride (TG) and total cholesterol (TC) levels	Decrease in white adipose tissues (WATs) and interscapular brown adipose tissue (iBAT) weight; promotion of WAT browning and thermogenesis	In adipose tissue: upregulation of fatty acid oxidation genes (*Cpt1B*, *Acox1*, *Pgc1*), thermogenesis-related genes (*Ucp1* and *Pparγ*) and markers of adipose tissue browning	Animal (mouse)	50 mg/kg (high-fat diet, HFD)	[41]
β-carotene	Preservation of liver functions	Regulation of lipid metabolism	Regulation of expression of genes involved in Pparα, Acly, and Fabp5 pathways in dorsolumbar and inguinal WAT (iWAT)	Animal (mouse)	150 mg/Kg (control diet)	[40]
		Promotion of thermogenesis in adipocytes	Activation of the β3-AR, cAMP, and α1-AR receptor pathways and increase in cytosolic Ca^2+^	Cell line (mouse 3T3-L1 preadipocytes)	20 μM	[111]
Vitamin A carrier RBP4	Induction of hepatic steatosis and mitochondrial dysfunction		Suppression of SIRT3-dependent long-chain acyl-CoA dehydrogenase (LCAD) deacetylation	Animal (transgenic mouse)	None (HFD)	[113]
	Promotion of de novo lipogenesis and lipid accumulation in hepatocytes and inflammation		Induction of M1-like polarization of Kupffer cells (KCs) by mediating the NOX2/ROS/NF-κB pathway	Cell lines (human Kupffer and hepatic LO2 cells)	25–100 ng/mL	[114]
				Animal (mouse)	50 μg/kg (HFD) Intravenous	
		Stimulation of basal lipolysis and inflammation	Increase of TNF-α production	Cell lines (human primary adipocytes and mouse macrophage RAW 264.7 cells)	50 µg/mL	[114]

## 4. Group B Vitamin

B vitamins, collectively known as B-complex vitamins, are eight water-soluble vitamins i.e., thiamine (B1), riboflavin (B2), niacin (B3), pantothenic acid (B5), pyridoxine (B6), biotin (B7), folate (B9), and cobalamin (B12). A possible link between some B vitamins (especially B3, B9, and B12) and NAFLD has been suggested, although a clear mechanism has not been well elucidated [117].

### 4.1. Vitamin B1

Vitamin B1 (thiamine) is a cofactor for enzymes involved in metabolic pathways such as glycolysis, the Krebs cycle, and the pentose phosphate pathways. Specifically, vitamin B1 acts as a coenzyme for the pyruvate dehydrogenase complex (PDC), α-ketoglutarate dehydrogenase complex (KGDHC), transketolase (TK), and the branched-chain ketoacid dehydrogenase (BCKDH) [118]. In particular, the vitamin B1-mediated activity of α-ketoglutarate dehydrogenase (α-KGDH), which is involved in regulating the turnover rate of the tricarboxylic acid (TCA) cycle, highlights the pivotal role of thiamine in the catabolism of carbohydrates and FAs [119].

Thiamine pyrophosphate (TPP) is the biologically active form of thiamine, produced by its intracellular phosphorylation. In weaned lambs fed a high-calorie (HC) diet, supplementation with thiamine reduces hepatic steatosis through microsomal triglyceride transfer protein (MTP), contributing to triglyceride (TG) depletion through very low-density lipoprotein (VLDL) secretion without affecting lipogenesis. Moreover, vitamin B1 decreases the expression of inflammatory genes and increases the expression of certain antioxidant genes (such as *SOD2*). As regards adipose tissue, interestingly, it was observed that TPP reduces adipose lipolysis; however, insulin resistance was not improved in weaned lambs [120] (Table 4).

Although no studies have specifically explored the connection between liver diseases and adipose tissue in relation to vitamin B1, thiamine supplementation has been observed to stimulate thermogenic markers in adipocytes. This may help inhibit obesity and enhance metabolic health [121].

### 4.2. Vitamin B2

Vitamin B2 is a critical component of coenzymes with various biochemical functions. Primarily, it serves as an essential component for flavin mononucleotide (FMN) and flavin adenine dinucleotide (FAD), which are involved in redox reactions intrinsically linked to energy metabolism. Specifically, FAD is involved in the Krebs cycle and the electron transport chain, while FMN is involved in dehydrogenation reactions, transferring hydrogen groups and their electrons from organic substrates to appropriate coenzymes. Thus, vitamin B2 generates energy by producing adenosine triphosphate (ATP) [122].

In addition to metabolic alterations, vitamin B2 is known to intensely affect lipid metabolism by controlling the expression of hepatic peroxisome proliferator-activated receptor γ (PPARγ). In diet-induced animal models of NAFLD, riboflavin deficiency is negatively correlated with PPARγ activity, leading to upregulated lipogenesis, dysregulated TG hydrolysis (by Adipose Triglyceride Lipase, ATGL), and impaired antioxidant mechanisms (including Glutathione Reductase (GR), superoxide dismutase (SOD), and glutathione peroxidase (GSH-Px) in the liver. Globally, the mentioned alterations might contribute to hepatic steatosis [123] (Table 4).

### 4.3. Vitamin B3

Vitamin B3 compounds, including niacin (nicotinic acid), nicotinamide (NAM), N1-methylnicotinamide (mNAM), and nicotinamide riboside (NR), serve as precursors of nicotinamide adenine dinucleotide (NAD) and nicotinamide adenine dinucleotide phosphate (NADP), which are known coenzymes involved in various metabolic reactions, including glycolysis, cellular respiration, fatty acid synthesis, and energy production through the citric acid cycle (Krebs cycle). Also, the NADPH/NADP rate maintains redox balance within cells and regulates the synthesis of fatty acids and steroids. In addition to the direct involvement of NAD+ in cellular metabolism, it also plays a role in cell signaling. This suggests that vitamin B3 may function at the intersection of metabolic regulation and cellular signaling. Overall, niacin plays a fundamental role in energy metabolism and regulation of lipid metabolism [124].

A recent study investigating the potential association between dietary vitamin B3 intake and NAFLD in 4378 affected patients found no clear linear association between a higher dietary niacin intake and a low risk of NAFLD. However, a stratified analysis revealed variations in the effect of niacin intake on NAFLD among groups with or without hypertension. The study concluded that a higher dietary niacin intake might be linked to a reduced likelihood of NAFLD in people with a healthy blood pressure range [125]. Interestingly, in humans, high vitamin B3 supplementation (12.3 mg/die) enhances the beneficial effects of lifestyle intervention on hepatic steatosis. The mechanism of this effect is not known; however, it may be due to its action on nicotinic acid receptor hydroxy-carboxylic acid (HCA) receptor 2 (HCA2) that may inhibit lipolysis in peripheral adipose tissue, reducing the flow of free fatty acids into the liver, thus playing a role in NAFLD pathogenesis [44].

In NAFLD-induced pathology by an obesogenic diet in rats, supplementation with vitamin B3 led to an increase in mitochondrial redox potential and a decrease in hepatic cholesterol content, preventing an increase in liver weight [126] (Table 4). However, other authors reported in a similar model that vitamin B3 did not cause a reduction in hepatic fat accumulation. Nevertheless, this supplementation proved effective in lowering serum TG and VLDL levels and enhancing insulin sensitivity [127]. Moreover, niacin showed anti-inflammatory effects on WAT of HFD-fed mice, partially mediated by adiponectin, whose gene and protein expressions are increased by niacin [128] (Table 4).

NAM administration has healing effects on diet-induced obesity in mice in adipose tissue and the liver. In BAT, NAM increases mitochondrial β-oxidation of FAs, and in WAT, it triggers a browning process, enhancing energy expenditure through a sirtuins-dependent mechanism. This metabolic activation is associated with anti-inflammatory effects by upregulating IL-10 and decreasing macrophage infiltration (CD68). NAM prevents hepatic steatosis by downregulating inflammatory (*Tnf-α*, *Ccl2*, and *Il6*) and fibrosis (*Col1a1* and *Mmp9*) gene expressions and inhibiting macrophage infiltration [45] (Table 4).

Under physiological conditions, mNAM triggers lipolysis in adipose tissue and gluconeogenesis in hepatocytes, releasing ketone bodies and glucose as metabolic substrates in skeletal muscle [46]. However, mNAM exhibits organ-specific effects in pregnant mice. It reduces hepatic accumulation by upregulating genes (*Pck1* and *G6pc*) related to gluconeogenesis in a NAD+/SIRT1-dependent manner, decreasing inflammation in WAT by downregulating *Il1b* and *Il6*, and increases oxidative stress in skeletal muscle, impairing glucose tolerance due to decreased GLUT4 [47] (Table 4).

Furthermore, NR has age- and sex-dependent healing effects on obesity-induced mixed M1/M2 inflammatory phenotype and collagen fiber remodeling in the gonadal WAT (gWAT) of old mice. These findings have potential implications for health outcomes, particularly in menopausal women, where NR could compensate for the lack of protection from obesity effects on the liver and WAT due to estrogen [48]. Additionally, NR shows promise in preventing high-fructose-induced lipid metabolism disorder by reducing lipogenesis in the liver and increasing lipolysis in WAT, suggesting its potential therapeutic use in lipid metabolic disorders [49] (Table 4).

### 4.4. Vitamin B5

Vitamin B5, also known as pantothenic acid, is the critical precursor of coenzyme A (CoA) biosynthesis. CoA is an essential cofactor involved in several biosynthetic pathways, including the synthesis of phospholipids, the synthesis and degradation of fatty acids, and the functioning of the TCA cycle and intermediary metabolism. Therefore, the limited bioactivity of vitamin B5 under nutrition deprivation can cause dysregulation of CoA-dependent metabolic pathways, such as the metabolism of lipids, leading to chronic metabolic disorders [129].

Metabolic dysfunction of NAFLD can stem from significantly dysregulated lipid and carbohydrate processes, causing the irreversible suppression of some enzymes involved in the biosynthetic pathway of coenzymes, including CoA [130,131]. Interestingly, the vitamin B5 deficiency-dependent inhibition of hepatic pantothenate kinase (PANK), which catalyzes the rate-limiting step in CoA synthesis, is not reversed by supplementation of vitamin B5, irreversibly resulting in hepatic lipogenesis. Moreover, vitamin B5 administration cannot overwhelm the activation of caspase-2, dependent on the CoA/acyl-CoA altered ratio, which contributes to NAFLD pathogenesis by increasing steatosis [130,131].

Recently, it has been shown that pantothenate, by targeting adipose tissue, promotes BAT energy expenditure in a UCP1-dependent manner, effectively preventing adiposity and, indirectly, hepatic steatosis [50] (Table 4).

### 4.5. Vitamin B6

Vitamin B6 encompasses six vitamers: Pyridoxine (PN), Pyridoxal (PL), Pyridoxamine (PM), and their respective phosphate esters: Pyridoxine 5′-Phosphate (PNP), Pyridoxal 5′-Phosphate (PLP), and Pyridoxamine 5′-Phosphate (PMP). It acts as a cofactor in over 150 enzyme reactions involved in amino acid metabolism, gluconeogenesis, and lipid metabolism [132,133].

Low plasma levels of PLP, the biologically active form of vitamin B6, are linked to cardiovascular disease, stroke, diabetes, and cancer [134]. Furthermore, vitamin B6 supplementation in NAFLD patients ameliorates hepatic fat deposits [135]. In accordance with this, it has been observed that vitamin B6 intake is inversely associated with hepatic steatosis; NAFLD patients typically have low vitamin B6 in the diet and low plasma levels [136]. Interestingly, high-fat diets in rats suggest that vitamin B6 supplementation exerts beneficial effects on the liver. More specifically, oral co-supplementation of selenium and vitamin B6 demonstrated a synergistic effect in hyperlipidemic rats by lowering liver lipid profiles (both lipid deposition and general steatosis) and decreasing the size of adipocytes in WAT. This was achieved by decreasing endogenous cholesterol and lipid formation, enhancing cholesterol transport to hepatocytes, and promoting FA β-oxidation [137] (Table 4).

### 4.6. Vitamin B7

Biotin, also termed vitamin B7, is primarily known as an antioxidant. Its physiological role is performed in the liver, where it participates as a carbon dioxide carrier to transfer a carboxyl group between the donor and acceptor in carboxylation reactions involved in gluconeogenesis, FA metabolism, and amino acid catabolism [138,139].

While no publications indicate a direct link between biotin and liver diseases, two recent papers highlight a connection between hepatic and adipose tissue and biotin, suggesting a possible role of this vitamin in liver diseases. It is reported that a high dose of biotin inhibits, in an AMPK-dependent manner, the expression of acetyl-CoA carboxylase β (*Acc2*) and proliferator-activated receptor γ coactivator 1α (*Pgc-1α*) genes, decreasing hepatic TG storage by enhancing mitochondrial β-oxidation [51]. Moreover, the combined supplementation of a prebiotic and biotin improves hepatic and adipose tissue metabolism [140] (Table 4).

### 4.7. Vitamin B9

Vitamin B9 (folate or folic acid) is an enzyme cofactor that is involved in different metabolic pathways, such as the methionine cycle and the trans-sulphuration pathway, purine and S-Adenosylmethionine (SAM) synthesis, epigenetic regulatory functions, and cellular redox status, through the regulation of one-carbon metabolism. In the plasma, folate is biochemically modified into the biologically active form 5-Methyltetrahydrofolate (5-MTHF), which enters enterocytes through the reduced folate transporter (RFC) and the high-affinity folate receptor (FOLR) for participating in folate-dependent reactions [141].

Observational studies identified a non-linear dose-response relationship between serum folate level and NAFLD [142,143]. Also, cross-sectional studies have demonstrated that higher serum folate levels are associated with lower odds of NAFLD; however, folate supplementation has poor therapeutic effects on NAFLD resolution [144]. Decreased hepatic expression of the folate transporter protein SLC19A1 led to a reduction of intracellular levels of folate acid, which triggers several hepatic mechanisms leading to NAFLD onset [145]. However, excessive or incorrect doses of folate can hinder fibrosis in NAFLD patients: during NAFLD fibrogenesis, HSCs undergo folate-mitochondrial metabolic switches activating the mitochondrial enzymes serine hydroxymethyltransferase 2 (SHMT2) and methylenetetrahydrofolate dehydrogenase 2 (MTHFD2), which are the bridge between mitochondrial folate metabolism and polyunsaturated FA metabolism [146]. In mice, dietary deficiency of choline and vitamin B9, vitamins preferentially targeting hepatic genes in a genetic background-dependent manner [147], is associated with changes in the hepatic miRNA expression patterns, regulating genes engaged in NAFLD pathophysiology. Some of these, such as miR-122, miR-34, and miR-21, have been previously identified in human NAFLD [148,149]. Vitamin B9 supplementation showed positive therapeutic effects since it reverses the pathogenic effects of miR-21 and miR-34 upregulation and miR-122 downregulation, possibly by restabilizing the methylation status of their corresponding genes *Hbp1*, *Sirt1*, and *Srebp-1c*, respectively [150] (Table 4).

In liver and adipose tissue, LDs accumulate due to the upregulation of lipogenic genes, and epigenetic inflammation responses worsen hepatic pathological features [145,151,152]. Interestingly, vitamin B9 can reverse adipose tissue hypertrophy, primarily affecting the route of transporter internalization. Moreover, it can negatively regulate the expression of genes involved in adipocyte proliferation, differentiation, and lipid accumulation, leading to an increase in specific metabolites (acetic acid, propionic acid, and isobutyric acid), and can modulate the gut microbiota, which in turn contributes to adipose tissue decrease, possibly through a feedback loop, and improve liver disease [52] (Table 4).

### 4.8. Vitamin B12

Vitamin B12 (cobalamin, Cbl) is an essential nutrient that is not synthesized in the human body but gained through dietary sources or supplements. Its uptake involves multiple steps: binding to intrinsic factor (IF) for absorption through a receptor-mediated mechanism in the enterocytes and subsequent modifications by various enzymes to convert B12 into its active forms, methylcobalamin (MeCbl) and adenosylcobalamin (AdoCbl). MeCbl and AdoCbl act as cofactors for two metabolic enzymes. In the cytosol, the folate-dependent cytosolic methionine synthase (MS) uses MeCbl as a cofactor to convert homocysteine to methionine in the methionine/S-adenosylmethionine (Met/SAM) cycle, leading to the methylation of biomolecules, which are fundamental for preserving cellular functions, and genomic stability. In the mitochondria, methylmalonyl-CoA mutase (MCM), using AdoCbl as a cofactor, converts methylmalonyl-CoA from odd-chain fatty acid β-oxidation to succinyl-CoA. Succinyl-CoA is part of the TCA cycle and heme biosynthesis, thus involved in cellular respiration and erythropoiesis [153,154,155]. Vitamin B12 is essential in the immune and nervous system functions, maintaining human gastrointestinal microbiota and regulating numerous other B12-dependent metabolic processes [156,157,158,159].

This complexity of adsorption and cellular internalization highlights the possibility that B12 deficiency, characterized by high serum levels of homocysteine and methylmalonic acid, despite inadequate intake, can be either due to its incomplete conversion into the active forms or due to its non-effectiveness in reaching the required cellular sites [6,159].

Vitamin B12 deficiency-dependent pathways leading to health issues have not been thoroughly elucidated, although alternative methylation and mitochondrial dysfunction caused by hyperhomocysteinemia (Hhcy) are identified as having a cause-and-effect relationship. Furthermore, vitamin B12 deficiency can depend on damage to the gut mucosa, decreasing intestinal absorption of vitamin B12 along with other essential vitamins (e.g., folate and vitamin B6) and giving rise to several physiological impairments [160,161,162].

Vitamin B12 deficiency upregulates de novo lipogenesis by activating the transcription factor SREBF1, and impairing β-oxidation of FAs [163]. Moreover, several human studies have revealed a negative correlation between insufficient vitamin B12 intake, consequently low serum levels, and NAFLD [4,164,165,166]. Specifically, a bidirectional interaction between NAFLD and vitamin B12 could generate a feedback loop encompassing the vitamin B12 metabolic pathway and those responsible for NAFLD pathogenesis. In addition, a meta-analysis study underscores the possibility that vitamin B12 and NAFLD share signaling pathways included in a feedback loop. Thus, their link may be a bidirectional cause-and-effect relationship, in which genetic variations associated with NAFLD determine dysregulation in the pathways controlling vitamin B12 metabolism, thereby increasing the serum vitamin B12 concentration and decreasing hepatic vitamin B12 levels, events leading to NAFLD onset, which in turn causes a further serum vitamin B12 increase and raises the risk for NAFLD [167]. Moreover, vitamin B12 can induce epigenetic changes in genes associated with hepatic lipid metabolisms through DNA methylation (DNAm) at CpG dinucleotides [168].

Vitamin B12-deficiency-dependent DNAm degree of selected cell-type-specific genes rewinds transcriptional programs, activating dysfunctional pathways, and bringing cellular shifts to hematopoietic tissues and the liver throughout NAFLD pathogenesis and hepatocarcinogenesis by further altering the expression of tumor-related genes [169,170].

From a therapeutic perspective, in addition to reverting the low rates of homocysteine methylation and transmethylation of methionine in NAFLD [171], vitamin B12 and folate dietary supplementation could restore homocysteine metabolism through the activation of Syntaxin 17 (STX17)-dependent autophagy, increasing β-oxidation of FAs and consequently decreasing inflammation and fibrosis [172] (Table 4).

Obesity, the most significant risk factor for hepatic lipid accumulation, is associated with a higher incidence of small intestinal bacterial overgrowth (SIBO), whose negative consequence on health is the malabsorption of several nutrients, including vitamin B12. Since lipid accumulation and increased intestinal permeabilization due to SIBO are related to NAFLD, vitamin B12 deficiency is a cofactor in its pathophysiology [4]. Indeed, there is a significant association between vitamin B12 deficiency and highly obese patients presenting dysfunctional lipid metabolism and hepatic steatosis [173]. Specifically, a recent study carried out on a large group of children, adolescents, and young adults revealed that lower vitamin B12 levels are linked to a higher body weight, increased adiposity, and poorer metabolic health [174]. However, meta-analysis and genetic studies reveal that the correlation between vitamin B12 deficiency and obesity can directly depend on patient factors, such as age-dependent gastrointestinal alteration, dietary patterns, gender, and genetic background [175,176].

**Table 4:** Key Mechanisms of B Vitamin’s Role in the Liver, Adipose Tissue, and the Liver-Adipose Tissue Axis. Gene and protein acronyms are reported at the end of the manuscript.

Compound	Effects on Liver	Effects on Adipose Tissue	Mechanisms	Models	Treatment	Reference
Vitamin B1	Reduction of hepatic steatosis, increased hepatic glycogen content		Increase of *MTP*, *PLIN2*, and *SOD2* gene expression; inhibition of TNF-α production	Animal (lambs)	300 mg/animal (high-calorie, HC, diet) Intravenous	[120]
		Increase of thermogenesis	Increase the expression of thermogenesis-related genes	Cell line (human primary adipocytes)	25 μM	[113]
Vitamin B2 deficiency	Alteration of lipid metabolism (with lipid accumulation) and antioxidant functions	Downregulation of ATGL	Upregulation of *FASN*, *CPT1*, and *PPARγ* protein expression; downregulation of *ATGL* expression; impaired antioxidant mechanisms, including GR, SOD, and GSH-Px	Animal (mouse)	Riboflavin deprivation, High-fat diet (HFD)	[123]
				Cell line (human hepatoma cell line HepG2)	0 and 3 nM	
Vitamin B3 (Niacin)	Regression of hepatic steatosis, reduction of cholesterol and triglyceride accumulation		Inhibition of hepatic gene and protein expression and activity of DGAT2	Animal (rats)	0.5% and 1.0% (HFD)	[126]
		Anti-inflammatory effect in epidydimal white adipose tissue (eWAT)	Partially through the increase of adiponectin expression	Animal (mouse)	360 mg/kg/d (HFD)	[128]
Vitamin B3 (nicotinamide, NAM)	Prevention of hepatic steatosis	Reduction of inflammation; shift into a brown-like phenotype; increase of mitochondrial β-oxidation of fatty acids (FAs) in inguinal white adipose tissue (iWAT). Reduction in lipid vesicle accumulation and increase of mitochondrial β-oxidation of FAs in interscapular brown adipose tissue (iBAT)	In the liver: downregulation of inflammatory (*Tnf-α*, *Ccl2*, and *Il6*) and fibrosis (*Col1a1* and *Mmp9*) gene expression. In adipose tissue: gene expression upregulation of the anti-inflammatory cytokine *Il-10*, *Ucp1*, NAD+ consuming enzyme *Sirt1*, genes involved in mitochondrial homeostasis (*Pgc1a* and *Pgc1b*, *Mfn2*, *Plin1* and *Cpt1b*) and genes involved in white adipose tissue (WAT) beiging (*Ppargc1a*, *Ppargc1b*, *Prdm16*); reduction of inflammatory (i.e., *Tnf-α*, *Il6*, and *Ccl2*) and fibrosis (i.e., *Col1a1* and *Mmp9*) gene expression. Increase of Ucp1 protein. Activation of AMPK. Decreasing macrophage infiltration	Animal (mouse)	1% (HFD)	[45]
Vitamin B3 (N1-methyl nicotinamide, mNAM)	Reduction of lipid accumulation	Reduction of inflammation in gonadal white adipose tissue (gWAT)	In liver: upregulation of genes (*Pck1* and *G6pc*) related to gluconeogenesis in a NAD+/SIRT1-dependent manner. In gWAT: downregulation of *Il1b* and *Il6*	Animal (pregnant mouse)	0.3 and 1% (HFD)	[47]
Vitamin B3 (nicotinamide riboside, NR)	Liver protection from obesity effects	Reduction in fat mass of gWAT and iWAT, reduction of inflammation and fibrosis in gWAT	In WAT: reduction of expression of macrophages markers (*Adgre1*, *Cd68)* and M1 macrophages genes (*Itgax*, *Tnf-α)*, M2 macrophages genes (*Mrc1*), and crow-like structures (CLS)	Animal (old female mouse)	400 mg/kg/d (HFD)	[48]
	Reduction of TG levels, fat deposition, and lipid synthesis. Anti-inflammatory effect	Increase of lipolysis in WAT	In liver: increase of NAD+/NADH redox imbalance and subsequent SIRT1/NF-κB pathway activation and IL-1β, IL-6, IL-18, and TNF-α downregulation; increase of FGF21 pathway activation. In WAT: Increase of FGF21 path activation	Animal (mouse)	400 mg/kg/d (high-fructose diet)	[49]
Vitamin B5	Indirectly, reduction of steatosis	Reduction of adipocyte lipide deposit in BAT, sWAT, and eWAT	Activation of BAT-inducing energy expenditure, and beige adipocyte promotion by phosphorylation of AMPK, which leads to induction of UCP1 expression by PGC1a	Animal (mouse)	10 mg/Kg (HFD)	[50]
				Cell line (human primary brown adipocytes)	1–5 mM	
Vitamin B6	Decrease of liver lipid deposition, moderating steatosis	Decrease of the adipocyte size in WAT	In liver: activation of hepatic mitochondrial β-oxidation by upregulation of the expression of liver lipase (*Hl*), *Sirt1*, and *Pparα*; inhibition of the lipogenesis pathway by decreasing the expression of *Srebp1c* and its downstream lipogenic enzymes *Acc* and *Fas*	Animal (rats)	2–3 mg/kg (HFD)	[137]
Vitamin B7	Reduction of hepatic triglyceride storage	Reduction of WAT weight	Activation of hepatic mitochondrial β-oxidation via upregulation of CPT activity; and inhibition of fatty-acid (FA) synthesis via downregulation of *Acc2*	Animal (rats)	37.9 mg/day (HFD)	[51]
Vitamin B9 deficiency	Steatosis		Genetic variations (SNPs) (rs1051266 and rs3788200) within SLC19A1 are associated with MALFD. SLC19A1-knockdown in the human cell line determines the downregulation of pathways controlling non-esterified fatty acid pathways, fatty amides, sterols, glycerophospholipids, and amino acid concentrations	Cell line (human liver THL2)	0.1 mg/ml	[145]
Vitamin B9	Impediment of fibrosis resolution		Activation of mitochondrial folate metabolism via upregulation of *Shmt2* and *Mthfd2* maintain profibrotic TGF-β1 signaling and polyunsaturated FA metabolism for hepatic stellate cells (HSCs) viable activation	Animal (mouse)	103 mg/Kg (normal chew)	[146]
				Cell lines (human LX-2 and mouse primary liver cell line)	10 mM	
	Normal hepatocytes in contact with sinusoids, central vein, and minimal number of apoptotic figures by impairing lipogenesis, insulin resistance, and imbalanced cytokine production		In a dose-dependent manner, restoring the physiological expression of hepatic miRNA via downregulation of miR-21 and miR-34, and upregulation of their related genes, *Hbp1* and *Sirt1*, respectively; and upregulation of miR-122 causes downregulation of *Srebp-1*	Animal (rats)	75 mg/kg (HFD)	[150]
	Decreased inflammation and fibrosis	Suppression of adipocyte proliferation, differentiation, and adipogenesis via downregulation of *IGF1*, *EGF*, and *TGF-*β	Increased intestinal folic acid transport carriers (RFC) is associated with the increment of Bacteroidetes (*Alistipes*, *Oscillospira*, *Ruminococcus*, *Clostridium*, *Dehalo-bacterium*, *and Parabacteroides*) and caecal short-chain fatty acids (SCFAs) (acetic acid, propionic acid, and isobutyric acid). Each caecal microbiota is positively correlated with the specific acetic acid content	Animal (broilers)	1.3 mg/kg (normal chew)	[52]
Vitamin B9 + Vitamin B12	Decreased inflammation and fibrosis		Impairment of STX17 proteasomal degradation recovers autophagy and restoration of homocysteine metabolism via upregulation of related genes (*Mat1a*, *Mthfr*, *Cbs*, *Mtr*, *Pon1*, *Pon2*, *Pon3*). Consequently, increased β-oxidation of FAs leading to decreased hepatic inflammation (IL6, IL1b, TNF-α) and chemokine (*Ccl2*, *Ccl5*, *Cxcl10*, *Cx3cl1*, *Cxcl16*) and fibrosis (*Tgf-*β, *Col1a1*, *Col1a2*, *Col3a1*, *Acta2*, *Ctgf)* genes	Animal (mouse)	B12 30 μg/~4700 kcal and Folate 6 μg/~4700 kcal (fructose in drinking water)	[172]

## 5. Vitamin C

Ascorbic acid (vitamin C) is crucial in maintaining lipid balance through its hypolipidemic effects. In HFD conditions, ascorbic acid reduces both serum and liver TGs, promoting lipolysis and decreasing MTP levels [177]. MTP is essential for transporting dietary and endogenous fats incorporated into apolipoprotein B (apoB) to other tissues [178]. Additionally, ascorbic acid enhances hepatic AMPK phosphorylation, thereby inhibiting the nuclear translocation of the liver X receptor (LXR) and suppressing genes involved in de novo lipogenesis (*SCD1*, *FASN*, and *SREBP-1c*) [177]. These pathways can be dysregulated in various metabolic disorders [179,180]. The mentioned findings suggest that ascorbic acid may interrupt the TG feedback loop in two interconnected ways crucial for managing hypertriglyceridemia, a condition known to be associated with the severity of NAFLD [181,182]. Pharmacologically, the prophylactic and therapeutic effects of vitamin C depend on the dose administered since unknown biochemistry mechanisms exist; only a medium dose of vitamin C maintains health by preventing the ill and healing effects of NAFLD [53]. Since the oxidative inflammatory cascade resulting from the interaction between mitochondrial and immune signaling is a significant event in NAFLD pathogenesis towards NASH, and since the supplementation of exogenous vitamin C has shown anti-inflammatory properties, a combination of vitamin C and an individual appropriate diet is used in clinical practice to delay NASH progression and improve clinical symptoms [183,184].

In the mouse model, vitamin C controls the hepatic expression and secretion of growth factor 21 (Fgf21) in a Pparα-dependent manner. The thermogenic hormone Fgf21 is translated into the adipose tissue, inducing BAT thermogenesis and regulating lipid metabolism [54] (Table 5). Fgf21 can regulate distinct metabolic pathways to control hepatic steatosis, inflammation, and fibrosis through lipid and carbohydrate metabolism [185,186]. Moreover, in human beings, dietary macronutrient distributions can differently influence the grade association between *FGF21* SNPs (single nucleotide polymorphisms) and NAFLD risks [187].

Vitamin C is also involved in epigenetic events, acting as a cofactor for the ten-eleven translocation (TET) family members (TET1, 2, and 3) that regulate DNA methylation patterns and epigenetic chromatin modifications [188]. Hypomethylation of adipogenesis-associated genes determines the hypertrophy of the adipocytes related to NAFLD pathogenesis [189,190]. Conversely, hypermethylation of the *PPARα* gene determines its downregulation in NAFLD patients. Consequently, the TET1 hypomethylating PPARα promotes fatty acid β-oxidation, impairing hepatic TG accumulation [191,192]. Genetic *TET* variants are linked to the development and progression of NAFLD toward NASH [169,193]. In mice, the supplementation of vitamin C, being involved as a cofactor for Tet enzymes [188], can impair WAT hyperplasia and hepatic steatosis in HFD induction, reversing the hypermethylation due to *Tet1* haploinsufficiency, leading to fatty acid oxidation lipolysis and thermogenic downregulation [55] (Table 5).

**Table 5:** Key Mechanisms of Vitamin C’s Role in the Liver, Adipose Tissue, and the Liver-Adipose Tissue Axis. Gene and protein acronyms are reported at the end of the manuscript.

Compound	Effects on Liver	Effects on Adipose Tissue	Mechanisms	Models	Treatment	Reference
Vitamin C	Modulation of gene expression	Induction of white-to-brown conversion, energy expenditure	In the liver: activation of the transcription factor Pparα leading to the secretion of thermogenic hormone Fgf21. In adipose tissue: Fgf21 controls thermogenic energy expenditure via *Ucp1* upregulation.	Animal (mouse)	2 g/L oral gavage, high-fat diet (HFD)	[54]
	Reduction of hepatic steatosis	Decrement of fat mass in epidydimal and inguinal white adipose tissue (eWAT and iWAT) and brown adipose tissue (BAT)	In iWAT: downregulation of lipogenic genes (*Srebf1*, *Fasn*, and *Acaca*) and upregulation of thermogenic genes (*Ucp1*, *ELovl3*, *Cox7a1*, *Dio2,* and *Cox8b*). In liver: upregulation of *Hsl*, *Ppara*, *Acox1*, and *Cpt1* and increases methylation of *Hsl* and *Ppara* promoters.	Animal (mouse)	0.36 g/kg (HFD)	[55]
			Hypermethylation of the HSL and PPARα promoters and upregulation of genes involved in fatty acid oxidation and lipolysis via reversion of TET1 haplo-insufficiency.	Primary human hepatocytes	200 mM	

## 6. Vitamin D

Vitamin D exists in endogenous forms (vitamin D3 or cholecalciferol) and dietary exogenous forms (vitamin D2 or ergocalciferol). The activation of these pre-hormones involves two tissue-specific hydroxylations. Initially, in the liver, they convert to 25-hydroxy vitamin D (25-OH-D) through the action of cytochrome P450 2R1 (CYP2R1). Subsequently, in the kidneys, 25-OH-D is further converted to its active form, 1,25-dihydroxyvitamin D (1,25(OH)_2_D), by cytochrome P450 27B1 (CYP27B1) [194].

The active form of vitamin D, 1,25-dihydroxyvitamin D (1,25(OH)_2_D), exerts pleiotropic effects by regulating target genes by binding to its nuclear vitamin D receptors (VDR). Inadequacy of 1,25(OH)_2_D is linked to obesity, attributed to the reduced expression of hydroxylase CYP2R1. This inadequacy is inversely correlated with TGs but not with other lipids (total cholesterol, low-density lipoprotein, LDL, and high-density lipoprotein, HDL), NAFLD, progression to NASH, and lobular inflammation [56,195,196].

Some evidence suggests that vitamin D deficiency contributes to NAFLD by engendering adipose tissue metabolism dysfunction, consequently altering the crosstalk between the liver and adipose tissue [57].

Vitamin D has a therapeutic potential effect on the liver-adipose tissue axis. It presents anti-inflammatory properties in adipocytes, suppressing nuclear factor kappa B(NF-κB) and mitogen-activated protein kinase (MAPK) signaling pathways through VDR-mediated mechanisms in adipocyte cells [197] (Table 6). Thus, it halts the progression of NAFLD to NASH mediated by the chronic inflammation of WAT [36,198].

Vitamin D deficiency contributes to NAFLD since it positively triggers WAT-associated macrophage infiltration. In turn, macrophages, secreting bioactive inflammatory adipokines (IL-6, TNF-α, and MCP1) active HSC, promote alerted extracellular matrix (ECM) remodeling, leading to fibrosis [58,199] (Table 6). However, dietary vitamin D supplementation could alleviate obesity-induced macrophage infiltration, their polarization to pro-inflammatory M1, and the production of pro-inflammatory related factors (e.g., TNF-α) [58].

Moreover, vitamin D supplementation inhibits HFD-dependent adipocyte hypertrophy by inducing autophagy, upregulating p53, and inactivating the PI3K/Akt/mTOR signaling pathways, smoothing the dysregulated lipid metabolism in adipose tissue [200,201] (Table 6).

Vitamin D supplementation blunts WAT inflammation, downregulating the related markers (*Mcp1* and *Ccl5*) and significantly decreasing de novo lipogenesis, downregulating the fatty acid synthase (*Fasn*) and acetyl-CoA carboxylase 1 (*Acaca*), leading to reduction of hepatic LDs in the liver but not in WAT. However, vitamin D alone cannot restore other dysmetabolic conditions, such as adiposity, insulin resistance, and glucose homeostasis [59] (Table 6). It must be mentioned that, in rats, vitamin D improves glucose homeostasis by promoting glycolysis versus gluconeogenesis [202].

Better outcomes can be achieved by combining distinct therapeutic strategies with the vitamin D cure. Indeed, vitamin D supplementation and physical exercises present straightforward synergetic effects on attenuating hepatic inflammatory genes *Tgfβ1* in WAT and *Mcp* in the liver. Since TGFβ1 and MCP are linked to regulating pathways involved in adipocyte lipogenesis and promoting insulin resistance, their downregulation reduces adiposity and restores insulin sensitivity. Thus, this synergism enhances HFD-related biochemical and inflammatory parameters [60,61] (Table 6). The vitamin D hepatoprotective property against steatosis is due to its capability to inhibit the NF-κB signaling in hepatic macrophages and KCs and decrease inflammatory cell infiltration [202,203].

Conversely to WAT, BAT activation enhances the clearance of circulating glucose, non-esterified fatty acids (NEFA), and TGs. However, NAFLD patients present lower BAT activity, which is inversely related to hepatic fat accumulation [204]. All of this underlies that BAT could be a therapeutic target. Interestingly, vitamin D at physiological concentration represses WAT differentiation and supports BAT differentiation, turning into rapid consumption of lipids [205] (Table 6).

Differences between calcifediol and calcipotriol on NAFLD have also emerged. Calcifediol presents pharmacokinetic properties that are more suitable for patients with liver failure or severe intestinal malabsorption since it is better absorbed, has higher biological activity, and is less prone to sequestration in adipose tissue [62]. Calcifediol increases VDR, and calcipotriol improves insulin sensitivity and hepatic steatosis [206,207]. However, a better understanding of the tissue-specific vitamin D function would strongly support the new therapy based on the prohormones of vitamin D [208].

In recent years, the diverse functions and regulatory processes of vitamin D in the pathophysiology and treatment of NAFLD and other chronic liver conditions have been attributed to its direct influence on miRNA expression. In NAFLD, circulating levels of miR-200c and miR-33a, pivotal in hepatic lipid metabolism, are initially downregulated during diet-induced fatty liver. However, their expressions are subsequently upregulated by vitamin D supplementation, thereby restoring physiological hepatic conditions through an unidentified mechanism [209]. Moreover, in eWAT, vitamin D can module the expression of inflammatory-linked miRNAs (miR-155, miR-146, and miR-150), thus downregulating the inflammatory signals in adipocytes by targeting the NF-κB signaling pathway [210,211,212]. Additional findings provide researchers with grounds for speculation regarding the potential role of vitamin D in the liver-adipose tissue axis in NAFLD. For instance, microbiota-derived gut metabolites influence the physicochemical characteristics of the gut microenvironment and modulate dysbiosis, which plays a fundamental role in the pathogenesis of obesity. Also, dysbiosis is associated with the progression or exacerbation of NAFLD and NASH [213]. It is noteworthy that vitamin D levels influence the profile and functionality of gut microbiota, which, in turn, with their metabolites, regulate the expression of the VDR [214].

Interestingly, individuals with obesity frequently exhibit lower circulating vitamin D levels due to its sequestration in adipose tissue [215]. Moreover, administering vitamin D in NAFLD patients ameliorates symptoms by enhancing specific microbiota-related metabolic pathways, thereby modulating hepatic metabolism [216,217] (Table 6).

It should be noted that vitamin D protects against NAFLD, ameliorating hepatic steatosis owing to obesity-induced dyslipidemia, by reducing plasma lipid uptake through fatty acid translocase (FAT/CD36) and increasing hepatic mitochondrial β-oxidation via the PPARα signaling pathway [218] (Table 6).

**Table 6:** Key Mechanisms of Vitamin D’s Role in the Liver, Adipose Tissue, and the Liver-Adipose Tissue Axis. Gene and protein acronyms are reported at the end of the manuscript.

Compound	Effects on Liver	Effects on Adipose Tissue	Mechanisms	Models	Treatment	Reference
Vitamin D		Inhibition of inflammatory pathway and adipokine expression	Anti-inflammatory activity: decrease of IL-6 and leptin protein expression through suppression of NF-kB and MAPK pathways via vitamin D receptor (VDR)	Human adipose tissue and adipocytes	10^−8^ M	[197]
	Protection from HFD effects	In epidydimal white adipose tissue (eWAT) suppression of adipogenesis, inflammatory responses, macrophage infiltration, and their phenotypic switch to M1 polarization	Inhibition of the transcription factor PPARγ and AP2, and decrease of the gene expression of *Tnf-α*, *Il-6*, and *Mcp* via inhibiting NF-kB inhibition and AMPK pathway activation	Animal (mouse)	1000 IU/kg (high-fat diet, HFD)	[58]
		Inhibition of browning of white adipose tissue (WAT)	Activation of p53 and inactivation of P13K/Akt/mTOR signaling leading to autophagy, impairment of brow-like adipocyte formation by downregulating the WAT browing markers (UPI1, PPARγ, PGCα)	Animal (mouse)	50 mg/kg (HFD)	[201]
				Cell line (mouse 3T3-L1 preadipocytes)	1–100 nM	
	Decrease lipid accumulation	Decrease inflammation	In the liver: suppression of de novo lipogenesis (*Fasn* and *Acaca*) and fatty acid oxidation (*Acox*)	Animal (mouse)	15,000 IU/Kg (high-fat/sucrose diet)	[59]
	Decrease inflammation and lipid accumulation	Decrease inflammation	In the liver: suppression of de novo lipogenesis (Fasn and Acaca) and chemokines Mcp1 In inguinal white adipose tissue (iWAT): strong suppression of *Ccl5* but slight suppression of *Tgfb1* and *Mcp1*	Animal (mouse)	15,000 IU/Kg (high-fat/sucrose diet)	[60]
	Enhancement in fatty degeneration	Prevention of hypertrophy of adipocytes	In liver: Reduction of FATP4 in liver. In liver and adipose tissue: decrease of TLR-4 in both liver and adipose tissue	Animal (rat)	500 IU/Kg (HFD)	[61]
		Enhancement of brown adipogeneis	Stimulation of brown adipogenesis program via *Prdm16* and *Pgc1α* upregulation and inhibition of white adipocyte differentiation via *Cebpb*, *Cebpa*, and *Pparγ* downregulation	Cell lines (mouse C3H10T1/2, 3T3-L1)	100 pM	[205]
	Reduction of fat vacuoles and inflammation		Inhibition of NLRP3 and pyroptosis, downregulation of ASC, cleaved-caspase-1, pro-IL-1β, IL-1β and GSDMD-N in liver tissues and BRL-3	Animal (rat)	5 mg/kg (HFD)	[217]
				Cell line (human hepatocellular HepG2)	10^−6^ mol/L	
	Enhancement of hepatic steatosis and systemic inflammation		In cells and rats, upregulation of β-oxidation by increasing expression of Pparα and Cpt1a and downregulation of fatty acid translocation (Fat/Cd36)	Animal (rat)	12.5 μg/Kg (HFD)	[218]
				Cell line (human hepatocellular HepG2)	25–200 nM	

## 7. Vitamin E

Vitamin E comprises eight lipid-soluble compounds, i.e., α-, β-, γ-, δ-tocopherol, β-, γ, and δ-tocotrienol. They have antioxidant activities, scavenge active oxygen radicals and oxygen, and protect from tissue damage, specifically, unsaturated lipids. Its principal site of action is in cell membranes and lipoproteins. Moreover, vitamin E stabilizes membrane lipid bilayers by forming complexes with membrane lipid components [219,220].

Oilseeds, nuts, fruit, and vegetables are the primary sources of vitamin E [221]. In the gastrointestinal tract, vitamin E is absorbed into enterocytes by the CM and HDL pathways and then transported to other tissues via circulation. Most vitamin E is metabolized in the liver [219].

Vitamin E modulates distinct transcription factors, including PPARγ, nuclear factor erythroid-derived 2 (NRF2), NF-κB, RAR-related orphan receptor α (RORα), estrogen receptor β (ERβ), and the pregnane X receptor (PXR) controlling gene expressions [222] encompassing several functions, such as liver metabolic homeostasis, fat absorption, immune system activation, among which physiological antioxidant activities. Furthermore, vitamin E plays a crucial role in regulating membrane fluidity, stability, permeability, and microdomains, including lipid rafts. Its ability to modulate membrane signal translations underscores its significance in disease prevention [223,224,225]. Despite various pieces of knowledge regarding the role of vitamin E in NAFLD, its definitive role is yet to be fully established due to inconclusive results in different studies [226,227].

The liver’s physiological and biochemical characteristics are intricately shaped by the presence of specific lipids, including α-tocopherol (α-TOH) and TG, along with their precise ratio. Disturbances in this ratio have the potential to profoundly influence the transcriptional regulation of genes, particularly those belonging to the cytochrome P450 (CYP) family responsible for hepatic lipid metabolism. Such perturbations can consequently lead to significant implications for the organ, impacting its healthful or unwholesome state. Specifically, the *CYP* gene response adheres to a parahormetic pattern within a healthy organ, as lipid stimulation remains below the physiological threshold. Conversely, if lipid stimulation exceeds this threshold due to the excessive accumulation of LDs, the *CYP* gene response transitions into pathophysiological conditions. This nuanced bimodal response of the *CYP* genes is intricately tied to hepatic homeostasis [228]. Additionally, the generation of LDs within hepatocytes further diminishes the amount of vitamin released into the sinusoids, resulting in impaired bioavailability [229]. In obesity-associated hepatosteatosis, the liver impounds α-TOH, diminishing the vitamin amount released into the sinusoids, thus decreasing the vitamin bioavailability to target tissues, resulting in pathophysiology.

Various studies have consistently confirmed that vitamin E effectively improves clinical outcomes in patients with NAFLD without positively enhancing total cholesterol and fibrosis scores [230]. Vitamin E achieves this by partially safeguarding the hepatocyte ultrastructure during hepatic steatosis, preventing the dilatation of the endoplasmic reticulum, blebbing of plasma membranes, and the cytoplasmic accumulation of LDs along with the engulfing of mitochondria [231].

Additionally, research indicates that during NAFLD, α-TOH diminishes TG accumulation by inhibiting hepatic de novo lipogenesis (DNL) through its antioxidant capacity and lipid solubility. This is achieved by impairing the post-transcriptional maturation of the DNL-gene transcription factor SREBP-1. However, disease treatment efficacy is not exceptionally high [232].

There is widespread awareness that combining various metabolites of different forms of vitamin E [219] or other distinct biochemical compounds [233] targeting different or the same pathways have synergistic and potential healing effects on NAFLD disease [234]. Indeed, α- and γ-tocopherol combination can have synergistic effects on adipose tissue and the liver in metabolic dysfunction. In combination, they can impair NF-κB signaling and activate PPARα, reverting lipid deposit and inflammation in adipose tissue and the liver [63]. Furthermore, α- and γ-tocopherol can decrease hepatic steatosis, modulating the expression of hepatic retinaldehyde dehydrogenases (*Raldh1*, *Raldh2*, and *Raldh3*), which govern the ubiquitous metabolism of retinol and the lipogenic factors SREBP-1c, which controls the synthesis of lipids from glucose in the liver [235].

The NAFLD progression is associated with the failure of the autophagy pathway, causing the accumulation of damaged organelles and consequent inflammatory steatohepatitis (HASH). Therefore, hepatic autophagy dysfunction can determine mitochondrial bioenergetic de-regulation, impairing metabolic flexibility. Indeed, in physiological conditions, hepatic mitochondria, which increases FA oxidation [236,237], and BAT peridroplet mitochondria (PDM), which upregulates TG synthesis in an oxidative phosphorylation (OXPHOS)-dependent manner [238], orchestrally curb intrahepatic accumulation of lipids preventing liver injury from lipotoxicity. However, the breaking down of this orchestra could lead to the hepatic accumulation of lipids and carbohydrates, seen as stress inducers, and hence mitochondria are subjected to metabolic remodeling from decreased to increased respiratory efficiency, which could determine the progression of the disease through the significant stimulus for hepatic DNL [239,240]. Recently, prolonged vitamin E supplementation has been demonstrated to disrupt interrelated miRNA profiles in the liver and WAT through negative feedback regulation, resulting in the upregulation of key transcription factors such as PGC1-α, SREBP-1/2, and Perilipin 1. This alteration subsequently impacts lipid metabolism in both the liver and WAT [64].

**Table 7:** Key Mechanisms of Vitamin E’s Role in the Liver, Adipose Tissue, and the Liver-Adipose Tissue Axis. Gene and protein acronyms are reported at the end of the manuscript.

Compound	Effects on Liver	Effects on Adipose Tissue	Mechanisms	Models	Treatment	Reference
Vitamin E	Reduction of steatosis inhibiting lipogenesis		Impairment of intrahepatic triglyceride (IHTG) accumulation by inhibition of maturation of the transcription factor SREBP-1, downregulating de novo lipogenesis genes (*FASN* and *SCD*)	Human (liver biopsies)	100 mmol/L	[232]
				Cell lines (human hepatocellular HepG2)		
Vitamin E (α- and γ-tocopherol)	Prevention of steatosis, oxidative stress, and inflammation	Reduction of adipocyte size and inflammation	In adipose tissue and the liver: inhibition of NF-κB nuclear translocation decreasing *Il-1β* and *Tnf-α* In the liver: positive modulation of Pparα, enhancing the expression of peroxisomal *Acox*	Animal (mouse)	0.7 mg/kg (high-fat diet, HFD)	[63]
Vitamin E	Impairment of lipid synthesis and activation of FA oxidation		Decreased expression of the dehydrogenases *Raldh1* and *Raldh2*, and the transcription factor *Srebp-1c*, inhibiting hepatic FA synthesis and transporter CD36	Animal (mouse)	0.7 mg/kg (HFD)	[235]
	Increase of triglycerides (TGs)	Moderated inflammation and mild increase in fat cell size	In the liver and white adipose tissue (WAT): dose-dependently suppresses the expression of *Pgc-1a* and *Srebp-1c* and Srebp-2c upregulation. In the liver and WAT: dose-dependent suppression of miRNAs (miR-22/miR-27) expression implicated in lipid metabolism	Animal (mouse)	100, 200, and 500 mg/kg oral gavage/day (normal chew)	[64]

## 8. Vitamin K

Vitamin K is a fat-soluble compound present in vegetables as phylloquinone (PK or VK1) and in fermented foods (cheeses) and meat as menaquinones (MK or VK2), which is classified into four subtypes (MK-4, MK-7, MK-8, and MK-9). Gut microbiota can synthesize this vitamin [241]. Each vitamin K isoform presents its food source-dependent absorption profile [242] and distinct tissue distribution and biological activity [243]. The vitamin K activity occurs in the endoplasmic reticulum through the vitamin K cycle, where it undergoes redox conversions catalyzed by two enzymes, vitamin K epoxide reductase (VKOR) and γ-glutamyl carboxylase (GGCX) [244]. The cycle catalyzes the post-translational carboxylation of glutamate residues to γ-carboxyglutamate (Gla) residues in several proteins (K-dependent proteins) involved in a variety of physiological and cellular processes, including blood coagulation, bone and soft tissue mineralization, signal transduction, and cell proliferation [245].

Vitamin K activity strongly depends on obesity since adipose tissue sequesters fat-soluble vitamin K in high-increased body fat conditions, leading to hypovitaminosis. Furthermore, the reduced vitamin K bioavailability increases insulin resistance and serum protein induced by vitamin K absence-II (PIVKA-II) [246]. The vitamin K-dependent serum increased levels of PIVKA-II, a serum biomarker for HCC [247], is one of the significant risk factors for the progression of NAFLD and it underlies the strong involvement of vitamin K deficiency in the NAFLD pathogenesis leading to HCC. Indeed, vitamin K deficiency supports liver injury in NAFLD, and the vitamin K protective effects depend on age; thus, it cannot reduce the risk for NAFLD in the elderly, probably due to its metabolism, which is directly related to age, sex, and hormonal homeostasis [136,248].

On HFD-induced NAFLD model mice, VK2 positively reduces the visceral fat burden without affecting the lean mass and free body fluid and prevents hepatic steatosis, inflammation, and fibrosis. Moreover, hepatic pathological changes are enhanced by relieving cholesterol metabolic disorder without improving dyslipidemia. However, vitamin K2 positively rewidens lipid metabolism disorders without affecting the K-dependent protein osteocalcin [65].

Recent research has focused on the activating role of vitamin K of the growth arrest-specific protein 6 (GAS6), revealing a rather complex mechanism. On the one hand, vitamin K supplementation is known to attenuate fat diet-induced hepatic steatosis by regulating the AMPK/SREBP1/PPARα signaling pathways via the activation of GAS6 [249]. On the other hand, GAS6, as a ligand of the TAM subfamily of receptor tyrosine kinases (Tyro3, Axl, and MerTK), can exert two contrasting effects on liver diseases: in acute liver injury, the GAS6/TAM axis promotes tissue repair and reduces inflammation, contributing to the wound healing response; conversely, in chronic liver diseases, it stimulates inflammation and tissue fibrosis [250]. Overall, this may support the progression from NAFLD pathogenesis to NASH and HCC by inflammation, immune and metabolic dysregulation, and fibrosis (via upregulation of fibrosis-related genes in an Axl/AKT-dependent manner), thus promoting an immunosuppressive and pro-tumor microenvironment [250,251].

**Table 8:** Key Mechanisms of Vitamin K’s Role in the Liver, Adipose Tissue, and the Liver-Adipose Tissue Axis. Gene and protein acronyms are reported at the end of the manuscript.

Compound	Effects on Liver	Effects on Adipose Tissue	Mechanisms	Models	Treatment	Reference
Vitamin K	Reduction of steatosis		Increase of AMPK phosphorylation, and downregulation of *Srebp1* and *Fas* and upregulation *Pparα*, *Cpt1a* and *Ucp2* via activating Gla-Gas6	Animal (mouse)	5 mg/kg (high-fat diet, HFD)	[249]

## 9. Conclusions

Our review underscores the significant role of vitamin deficiency in the intricate crosstalk between the liver and adipose tissue. The findings suggest that vitamin deficiencies, through various molecular pathways, alter the liver-adipose tissue axis and can disrupt energy balance (with specific reference to lipid metabolism) and affect inflammation, leading to steatosis in the liver and the subsequent development and progression of conditions such as NAFLD and NASH.

**Figure 3:**
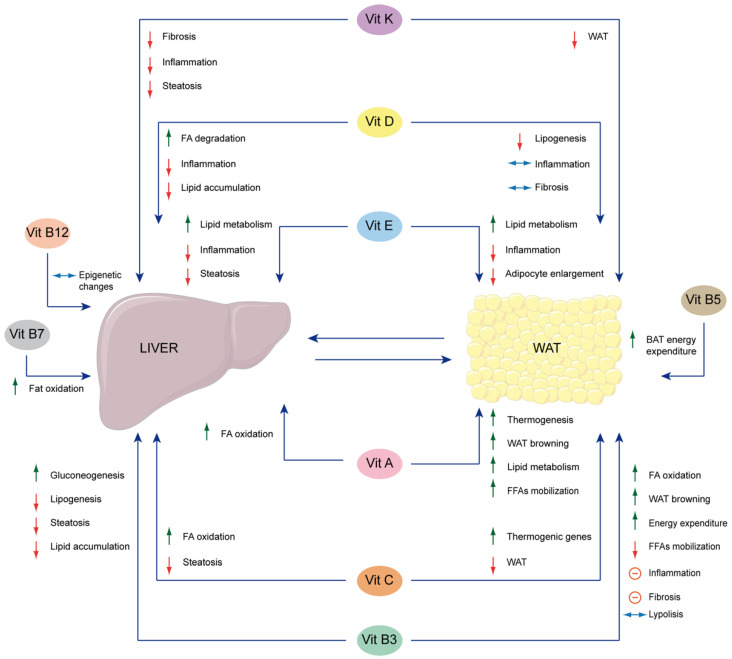
Effect of vitamins on the liver-adipose tissue axis. This figure highlights the most well-characterized mechanisms discussed in this review regarding the impact of vitamins on the liver-adipose tissue axis. Vitamins with arrows directed solely towards the liver or adipose tissue indicate that their primary effects are concentrated on the specified organ. However, it is understood that these effects also influence the other organ, contributing to the overall progression of liver disease. Legend: ↑ = Increase ↓ = Decrease ⊝ = Inhibition ↔ = Modulation. This image was created using Inkscape.

In this scoping review, after selecting 183 eligible papers from the PubMed database search, we identified 31 articles as relevant to the focus of this review, which were then categorized into groups based on the types of vitamins. The comprehensive analysis of these studies reveals that metabolic pathways and inflammation are the most frequently studied areas, or at least the most prominent ones that emerge in this type of research. Many of the studies examined in this review report correlations among clinical data, alterations in the liver and adipose tissue, and vitamin deficiencies. However, some studies go further to identify specific cellular and molecular mechanisms. Figure 3 provides an overview of the mechanisms involved in the liver-adipose tissue axis discussed in this review. For instance, vitamin D modulates inflammatory responses in adipose tissue, and vitamins E and C control lipid metabolism in adipose tissue and the liver through miRNA [211]. Moreover, vitamin B12 can induce epigenetic changes in genes associated with hepatic lipid metabolisms through DNA methylation [168]. Also, it is important to stress that while several pieces of evidence provide clues about the role of vitamins in the liver and adipose tissue, thus speculating an alteration in the liver-adipose tissue axis, these must be confirmed by specific studies, for example, considering that vitamin B1 can reduce hepatic steatosis [120] and improve thermogenic markers in adipocytes [121]. These observations lend plausibility to the hypothesis that vitamin B1 treatment could improve the overall clinical picture through its combined effects on both liver and adipose tissue, despite the lack of direct research linking them.

While the reciprocal relationship between the liver and adipose tissue is well-known when examining vitamin deficiencies (e.g., hepatic EV-carrying miRNA affecting lipogenesis and inhibiting lipid oxidation in adipose tissue), it is evident that existing studies primarily focus on the impact of adipose tissue on the liver. The reverse relationship remains less explored, highlighting a critical gap in our understanding and suggesting that further research is needed to fully elucidate these dynamics.

Given the crucial role that vitamins play in regulating enzymatic processes within the liver and adipose tissue and the clear evidence that liver diseases are metabolic diseases, they are emerging as promising therapeutic targets. Addressing vitamin deficiencies, either alone or in combination with other therapeutic strategies, holds potential for reversing dysfunctions in adipose tissue and the liver, thereby offering new avenues for managing NAFLD.

Our review consolidates evidence from research conducted between 2019 and 2024, emphasizing the need for a deeper exploration of the potential cause-effect dynamics between the liver-adipose tissue axis and vitamin deficiency in liver disease. This approach could pave the way for novel therapeutic interventions to mitigate the impact of vitamin deficiencies on liver health.

## Gene and Protein Abbreviations

ACACA	Acetyl-CoA Carboxylase 1
ACC2	Acetyl-CoA carboxylase beta
Acly	ATP-citrate Lyase
Acox1	Acyl-CoA Oxidase 1
Acta2	Actin alpha 2, smooth muscle
AFABP	Adipocyte Fatty Acid-Binding Protein
Akt	Protein kinase B
AMPK	AMP-activated Protein Kinase
aP2	Adipocyte protein 2
apoB	Apolipoprotein B
ASC	PYD and CARD domain containing
ATGL	Adipose Triglyceride Lipase
ATP	Adenosine Triphosphate
BCKDH	Branched-Chain Ketoacid Dehydrogenase
BCMO1	β-carotene 15,15′-monooxygenase 1
BRL-3	BRI1-like 3
Cbs	Cystathionine beta-synthase
CCDC80	Coiled-Coil Domain Containing 80
Ccl2	C-C Motif Chemokine Ligand 2
Ccl5	C-C Motif Chemokine Ligand 5
Cebpa	CCAAT enhancer binding protein alpha
Cebpb	CCAAT enhancer binding protein beta
Col1a1	Collagen type I alpha 1 chain
Col1a2	Collagen type I alpha 2 chain
Col3a1	Collagen type III alpha 1 chain
COL6A2	Collagen type VI alpha 2 chain
Cox7a	Cytochrome c oxidase subunit 7A1
Cox8b	Cytochrome c oxidase subunit 8B
Cpt1	Carnitine O-palmitoyltransferase
CPT1A	Carnitine palmitoyltransferase 1A
Cpt1B	Carnitine palmitoyltransferase 1B
Ctgf	Connective tissue growth factor
CX3CL1	C-X3-C motif chemokine ligand 1
CXCL10	C-X-C motif chemokine ligand 10
CXCL16	C-X-C motif chemokine ligand 16
CYP	Cytochrome P450
CYP27B1	Cytochrome P450 27B1
CYP2R1	Cytochrome P450 2R1, Vitamin D 25-Hydroxylase
Dio2	Iodothyronine deiodinase 2
DUSP1	Dual-specific phosphatase
EGF	Epidermal growth factor
ELOVL3	ELOVL fatty acid elongase 3
ERβ	Estrogen receptor beta
Fabp5	Fatty Acid Binding Protein 5
FAD	Flavin Adenine Dinucleotide
FASN	Fatty Acid Synthase
FATP4	Fatty acid transport protein-4
FGF21	Fibroblast Growth Factor 21
FMN	Flavin Mononucleotide
G6pc	Glucose-6-phosphatase catalytic subunit
GADD45B	Growth arrest and DNA-damage-inducible 45 beta
GGCX	γ-glutamyl Carboxylase
GAS6	Growth Arrest-Specific protein 6
GLUT4	Glucose transporter type 4
GR	Glutathione Reductase
GSDMD-N	Gasdermin D
GSH-Px	Glutathione Peroxidase
HBP1	HMG-box transcription factor 1
HDL	High-density lipoprotein
Hsl	Hormone-sensitive lipase
IGF1	Insulin-like growth factor 1
IL-10	Interleukin-10
Il-1β	Interleukin-1 beta
IL-6	Interleukin-6
IL-8	Interleukin-8
KGDHC	α-Ketoglutarate Dehydrogenase Complex
LCAD	Long-chain acyl-CoA dehydrogenase
LDL	Low-density lipoprotein
MAPK	Mitogen-activated Protein Kinase
Mat1a	Methionine adenosyltransferase 1A
MCM	Methylmalonyl-CoA mutase
MCP1	Monocyte Chemoattractant Protein 1
Mmp9	Matrix metalloproteinase 9
MS	Methionine synthase
MTHFD2	Methylenetetrahydrofolate dehydrogenase 2
Mthfr	Methylenetetrahydrofolate reductase
mTOR	Mammalian target of rapamycin kinase
MTP	Microsomal Transfer Protein
Mtr	5-methyltetrahydrofolate-homocysteine methyltransferase
NADP	Nicotinamide Adenine Dinucleotide Phosphate
NADPH	Nicotinamide Adenine Dinucleotide Phosphate
NFκB	Nuclear factor kappa B
NLRP3	NLR family pyrin domain containing 3
NOX2	NADPH Oxidase 2
NRF2	Nuclear factor erythroid-derived 2
PANK	Pantothenate Kinase
Pck1	Phosphoenolpyruvate Carboxykinase 1
PDC	Pyruvate Dehydrogenase Complex già esteso
Pgc1	Peroxisome Proliferator-activated Receptor Gamma Coactivator 1
PGC1-α	Peroxisome Proliferator-activated Receptor Gamma Coactivator 1-alpha
PI3K	Phosphatidylinositol 3-kinases
PIVKA-II	Protein Induced by Vitamin K Absence-II
Pon1/2/3	paraoxonase 1/2/3
PPAR-α	Peroxisome Proliferator-Activated Receptor Alpha
PPARγ	Peroxisome Proliferator-Activated Receptor Gamma
Prdm16	PR domain containing 16
PXR	Pregnane X receptor
RALDH 1/2	Retinaldehyde dehydrogenases 1/2
RBP4	Retinol-Binding Protein 4
RFC	Reduced folate transporter
RORα	RAR-related orphan receptor alpha
SCD	Stearoyl-CoA Desaturase
SCD1	Stearoyl-CoA Desaturase 1
SHMT2	Serine hydroxymethyltransferase 2
Shmt2	Serine hydroxymethyltransferase 2
SIK1	Salt inducible kinase 1
SIRT	Sirtuin
SIRT1	Sirtuin 1
SLC19A1	Solute carrier family 19 member 1
SOCS3	Suppressor of cytokine signaling 3
SOD	Superoxide Dismutase
SOD2	Superoxide Dismutase 2
SOD3	Superoxide Dismutase 3
SREBF1	Sterol regulatory element-binding transcription factor 1
SREBP-1	Sterol regulatory element-binding protein 1
SREBP-1c	Sterol regulatory element-binding protein 1c
Stx17	Syntaxin 17
TET	Ten-Eleven Translocation enzymes
TGF-β1	Transforming Growth Factor-beta 1
TK	Transketolase
TLR-4	Toll-like receptor 4
TNF-α	Tumor Necrosis Factor-alpha
TPP	Thiamine Pyrophosphate
TTR	Transthyretin
UCP1	Uncoupling Protein 1
VKOR	Vitamin K Epoxide Reductase
VLDL	Very Low-Density Lipoprotein
α-KGDH	Alpha-ketoglutarate dehydrogenase
Adgre1	Adhesion G protein-coupled receptor E1
CD68	Cluster of Differentiation 68
Mrc1	Mannose Receptor C-Type 1
